# Nutrition and Lifestyle Behavior Peer Support Program for Adults with Metabolic Syndrome: Outcomes and Lessons Learned from a Feasibility Trial

**DOI:** 10.3390/nu12041091

**Published:** 2020-04-15

**Authors:** Muhammad Daniel Azlan Mahadzir, Kia Fatt Quek, Amutha Ramadas

**Affiliations:** Jeffrey Cheah School of Medicine and Health Sciences, Monash University Malaysia, Jalan Lagoon Selatan, Bandar Sunway 47500, Malaysia; quek.kia.fatt@monash.edu

**Keywords:** metabolic syndrome, peer support, diet, lifestyle, intervention, feasibility

## Abstract

Background: While peer support interventions have shown to benefit adults with certain chronic conditions, there is limited evidence on its feasibility and effectiveness among people with metabolic syndrome (MetS). This paper describes the outcomes of a pre-post feasibility trial of “*PE*e*R* *SU*pport program for *AD*ults with m*E*tabolic syndrome” (PERSUADE), an evidence-based and community-specific nutrition and lifestyle behavior peer support program for Malaysian adults with MetS. Methods: We recruited 48 peers (median age: 46 (IQR = 11) years old) into four peer groups, who underwent 3 months of PERSUADE, followed by 3 months of follow-up period. Statistical analyses were conducted at post-intervention and post-follow-up to assess the changes in nutrition intake, anthropometry, and metabolic parameters. Results: Although there were significant overall increases in total carbohydrate intake and glycemic load (both *p* < 0.001), we noted significant reductions in the intakes of total energy and fat (both *p* < 0.001). Physical activity (total METS/week) also showed a significant improvement (*p* < 0.001). Overall, significant but marginal improvements in anthropometric and vital metabolic parameters were also observed. Conclusions: The feasibility trial supported the adoption of PERSUADE, though there is a need to assess the long-term impact of the peer support program in local community settings.

## 1. Introduction

Metabolic syndrome (MetS) is a clustering of metabolic risk factors that includes abdominal obesity, elevated serum levels of triglyceride (TG), fasting blood glucose (FBG), raised blood pressure (BP), but a reduced level of high-density lipoprotein (HDL). These changes may increase the risk of developing type 2 diabetes mellitus (T2DM) and cardiovascular diseases (CVD) [1]. Multiple nationwide cross-sectional studies on MetS in Malaysia have suggested that the prevalence falls between 26.5% to 43.4% depending on definitions used in the study [2]. This alarming prevalence may be due to changes in economic development, modernization, and urbanization in developing countries such as Malaysia [3].

Similar to other lifestyle-related chronic diseases, MetS is closely related to poor nutrition and dietary profiles, as well as sedentary lifestyle [2]. It is important for MetS to be identified early, not only because of the associated risks but also to put forward lifestyle as the initial focus of therapy. Components of MetS have been shown to improve with positive changes in diet and physical activity levels [4]. The National Cholesterol Education Panel Adult Treatment Panel III (NCEP-ATP) [5] recommended lifestyle modification as the first strategy in MetS management, which is also in line with Malaysian National Non-communicable Diseases Planning 2010–2015 [6]. Furthermore, short-term and intensive lifestyle intervention targeting weight loss among adults with MetS is effective at improving the clinical outcomes [7]. However, the literature suggests a high possibility of relapse over time, where participants regained weight loss or exhibit worse markers of MetS [8]. 

Peer-led intervention programs have been suggested to address lifestyle-associated non-communicable diseases in other parts of the world [9]. The effectiveness of peer intervention has also been previously assessed among female factory workers with MetS in the past [10]. Several studies assessed the effectiveness of lifestyle interventions to modify the health behaviors of the group affected by components of metabolic disorders in Malaysia, such as T2DM [11], gestational diabetes [12], and dyslipidemia [13], but not the clustering effect as in MetS. Under the National Strategic Plan for Non-Communicable Diseases in Malaysia, the Ministry of Health mobilized existing healthcare facilities to actively promote public health awareness and advocates a national policy on healthy diets and physical activity [6]. However, because there is no consensus for the MetS disease status, the strategic plan only targets metabolic diseases individually regardless of clustering.

To the best of our knowledge, no published experimental peer-led intervention related to MetS has been reported in Malaysia. The intervention conducted in Malaysia primarily incorporates functional foods as supplements in addition to lifestyle changes to manage MetS [2]. Besides, reported lifestyle interventions on non-communicable diseases in Malaysia put little emphasis on peer support components except for mental health-related studies. PERSUADE (nutrition and lifestyle behavior *PE*e*R SU*pport program for *AD*ults with m*E*tabolic syndrome) was developed as a result of the growing evidence on the peer-led framework on chronic diseases [9]. The development of PERSUADE incorporated systematic steps to ensure it was relevant, community-specific, and evidence-based. PERSUADE aimed to educate adults with MetS with the knowledge of MetS, its risk factors, and the importance of preventative measures such as increased physical activity and improved diet quality. Here, we provide a brief description of PERSUADE and report the effect of the program on dietary and lifestyle behaviors, as well as the anthropometric and metabolic parameters.

## 2. Materials and Methods 

### 2.1. Development of PERSUADE Peer Support Module 

PERSUADE was developed and tested for effectiveness based on a 5-step approach; (1) review of published MetS literature; (2) focus group discussion (FGD) among adults with MetS; (3) behavior change matrix incorporating findings from the review, FGD, and Health Belief Model (HBM); (4) program modules development; and (5) feasibility trial.

In the 1st step, evidence synthesis was conducted on available literature on peer-based lifestyle intervention on the metabolic syndrome to systematically assess published peer group-based lifestyle intervention targeted for MetS. Subsequently, in the 2nd step, a series of focus group discussions were conducted among a group of Malaysian adults with MetS (n=21) to qualitatively explore their understanding of MetS and their perceived motivation and barriers to healthy nutrition and lifestyle behavior.

In the 3rd step, a behavioral change matrix was prepared to incorporate both findings from the qualitative synthesis (Step 2) and the behavioral recommendations obtained from evidence synthesis (Step 1). This matrix was enhanced using the theoretical constructs of the HBM [14] and became the backbone framework of peer module content development. A 12-weeks module, including training modules for peer leaders and peers, was designed based on the behavioral change matrix in the 4th step. 

A detailed description of the development and process evaluation of PERSUADE has previously been published [15]. The final step, which was a feasibility trial of PERSUADE, is explained in Section 2.2.

### 2.2. Feasibility Trial 

The effect and feasibility of PERSUADE were assessed via a pre-post trial conducted between February and September 2019. The feasibility trial consisted of 2 parts: The 1st part involved peer leader training for 2 days, and the 2nd part involved a series of peer sessions implemented by peer leaders over a period of 3 months. The peers were then followed-up for another 3 months after completion of the intervention. During follow-up periods, no intervention was provided to the participants.

**Figure 1:**
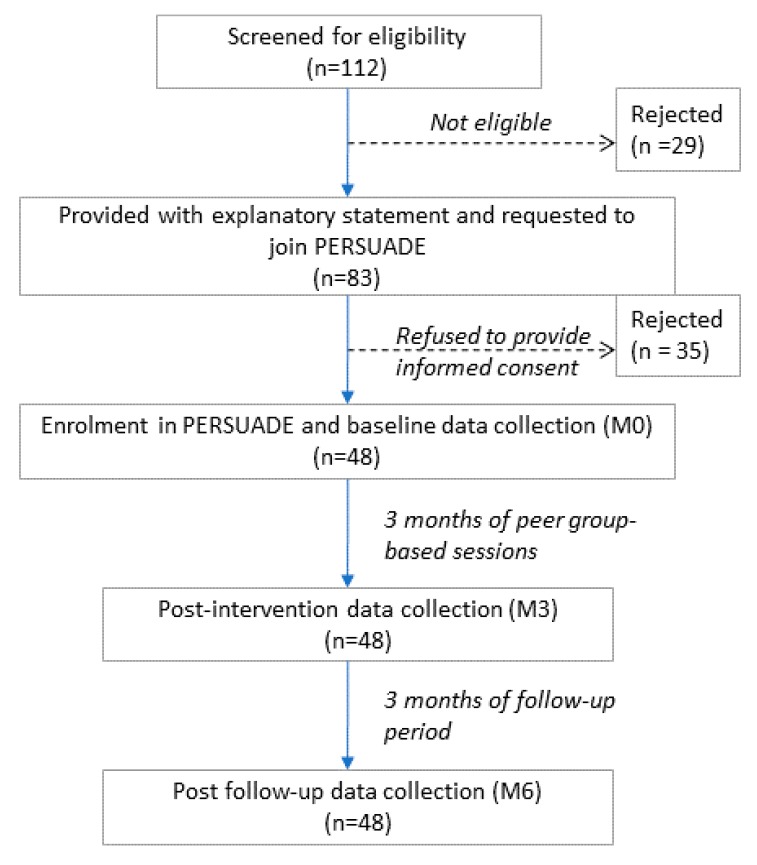
Study flow chart.

The trial took place in 2 separate neighborhoods in the district of Kulai, Taman Skudai and Taman Johor Jaya, in the state of Johor. In both neighborhoods, a recruitment drive was done to screen adults with MetS based on the harmonized criteria for MetS [3]. Ethical approval was obtained from the Monash University Human Research Ethics Committee (MUHREC) (CF16/56—2016000022) before the commencement of the study. Figure 1 presents the feasibility study flow chart.

#### 2.2.1. Study Participants

Recruitment drives were done in 2 neighborhoods through health screening programs organized by the respective neighborhood committees. The committees also helped spread the word on the health screenings among their communities. All interested community members who attended the health screening were also screened according to the study’s eligibility criteria, and eligible participants were invited to join the study. In order to be eligible, volunteer participants had to be Malaysian and fulfilled MetS according to the harmonized criteria [3]: 1) Waist circumference (WC) (female ≥ 80 cm; male ≥ 90 cm), 2) raised triglyceride (TG) levels (≥1.7 mmol/L), 3) reduced HDL cholesterol (female < 1.0 mmol/L; male < 0.9 mmol/L), 4) raised blood pressure (BP) (systolic ≥130 mmHg or diastolic ≥85 mmHg), or 5) raised fasting plasma glucose (FBG) (≥5.6 mmol/L). 

In addition, participants had to be willing and able to commit to a 12-week peer module. Participants were ineligible to participate if they were reported to have cardiovascular diseases, chronic liver or kidney disease, thyroid problems, and advanced cancer. 

An audit of pilot studies reported a median of 36 participants in feasibility trials [16]. Hence, we aimed to recruit at least 44 participants after adding an additional 20% to account for potential dropouts to the reported median sample size. We successfully enrolled 48 participants into the study at the end of the recruitment process.

#### 2.2.2. Peer Leader Training Program

The 48 participants were divided into 4 groups of 5 to 8 members according to the location of their neighborhoods to maximize participation. Each group was led by 1 volunteer peer leader. Peer leaders had to fulfill the following criteria; ability to communicate verbally, available for all 12 weeks of study, and willing to attend 2 days of peer leader training. Four peer leaders were appointed and were responsible for all participants in 4 peer groups, respectively. The training course for peer leaders involved; (1) brainstorming and discussion on motivation and barriers on behavioral change; (2) introduction to the PERSUADE peer module; and (3) self-efficacy skills workshop. The total training course was 16 hours long and comprised of 4 knowledge sessions on MetS, good dietary habits, physical activity, and healthy lifestyle behaviors (sleeping pattern, smoking, hydration, medication adherence, and supplement intake). The training also involved 4 workshops on self-efficacy skills (body weight, blood pressure and waist circumference measurement, dietary record, physical activity record, and reading food labels).

#### 2.2.3. Peer Session by Peer Leaders

As a part of the 3-months peer-led intervention, each participant was provided with a PERSUADE peer handbook that consisted of posters and peer activity guide on MetS, physical activity, healthy diet, and lifestyle behavior. The handbook also allowed participants to monitor and record their weight, physical activity, and dietary intake, as shown by peer leaders at respective group sessions. The intervention for week 1 started with a large group activity where 4 peer leaders, together with the research team, conducted a 2-hours introduction and peer support session. All participants had activities for their own group to set their knowledge and understanding goals and health goals for the rest of the program. 

The large group activity was followed by a 2nd peer session led by the 4 peer leaders individually in small-groups, and this format was sustained until the end of the program. The weekly small-group peer sessions of 60 minutes duration were organized by each group’s peer leader to strategize steps on achieving significant changes in lifestyle behavior, dietary intake, physical activity levels, and weekly body weight. Each support session was designed to improve peer social support through facilitated group discussion and learning by sharing experiences and problem-solving strategies with each other. At the start of each peer session, participants measured their body weight in order to set goals to be achieved at their next meeting. Each time, a target goal was set with participants relating to their body weight, dietary intake, and physical activity levels. At the end of the 12th week, the research team identified the most successful group who lost the most weight to discuss and share experiences with all peer groups, particularly on their challenges and how they overcame barriers faced throughout the study period. 

#### 2.2.4. Data Collection and Measurements

Data were collected from participants at recruitment (baseline), at the end of the feasibility trial (3 months post-enrollment), and again after 3 months follow-up (6 months post-enrollment) during face-to-face sessions with the study nutritionist, to assess the changes in nutritional, lifestyle, anthropometry and metabolic parameters across the 3 time points. Anthropometry measures, including height and waist circumference, were measured according to the World Health Organization protocol [17] using the SECA stadiometer. Weight and body fat percentage (BF) were measured using InBody 120 Body Composition Analyzer. Body Mass Index (BMI) was calculated as weight in kilograms divided by height in meters squared. BP was assessed using OMRON HEM-907XL automated BP monitor. Furthermore, FBG and TG and HDL-cholesterol were measured via the finger-prick method. Both B Braun Omnitest 3 Glucometer (FBG) and Cardiochek PA Blood Meter (TG and HDL) were calibrated and validated for screening.

Dietary data were collected using a 24-hour dietary recall at all 3 time points described above. The dietary recall included probing for information on the mealtime of the day, food preparation methods, and portion size of each food and beverage. We used a validated visual aid [18] to help participants report accurate portion sizes. Nutritional data were analyzed using DietPLUS Version 3 [19,20]. A short questionnaire was used to gather information on dietary and lifestyle behaviors, including the number of meals consumed, eating speed, late-night eating, breakfast-skipping, dining out, supplement intake, smoking habits, and sleeping duration. Physical activity levels were measured using the validated short-form International Physical Activity Questionnaires (IPAQ) [21], and data were converted to metabolic equivalent task minutes per week (MET-minute/week). 

#### 2.2.5. Data Analysis

The study population was described using the median, interquartile range (IQR), mean, standard deviation (SD), frequency, and percentage. Characteristics of peers were compared across the peer groups using Kruskal Wallis or Fisher’s Exact test to ensure that there were no statistical differences in demographic characteristics between the peer groups. The normality of continuous variables was determined using the Shapiro Wilk test. Total fiber intakes, DBP, FBG, BF, and TG, were not normally distributed, and natural log transformations were performed. Repeated measures were used to compare the changes in nutritional, anthropometric, and metabolic parameters. If there was an overall statistically significant difference between the time points, Bonferroni pairwise comparisons were performed. Magnitudes of change in continuous variables (d) were calculated to demonstrate the immediate and sustained effects of the intervention. Categorical variables were compared between similar time points using the McNemar test. All statistical analyses were performed with IBM SPSS Statistics 25.0, and the statistical significance was set at *p* < 0.05.

## 3. Results

The characteristics of participants are presented in Table 1 according to their peer groups. None of the characteristics differed according to the groups they were assigned. The total number of participants was 48; 23 (47.9%) males and 25 (52.1%) females. All participants successfully completed the trial. The level of adherence was high, as most (81.3%) of the participants attended the peer group session. While 6.3% and 12.5% attended 10 and 11 peer sessions, respectively, and most of the absences were due to work commitments and family issues.

### 3.1. Dietary Behaviors

Table 2 presents the changes in nutritional parameters assessed in the feasibility study. We found reductions in total energy intake at post-intervention (d = −4.15%, *p* = 0.045) and at post follow-up (d = −9.90%, *p* = 0.015). We hypothesized the overall reduction of total fat intake (*p* = 0.001) to contribute towards the decrease in total energy and masked the changes in carbohydrate and protein intakes. Interestingly, we also found a significant increase in energy-adjusted total fiber intake at post follow-up (d = +30.36%, *p* < 0.001) and almost equal percentage of increase in total sugar per 1000 kcal (d = +39.06%, *p* = 0.002). The glycemic load showed an increase at post-intervention (d = +14.93, *p* < 0.001), but the increase stabilized over the follow-up period. The unexpected changes in total sugar and glycemic load were very likely due to an increased intake of fruits, as recommended in the PERSUADE program module.

**Figure 2:**
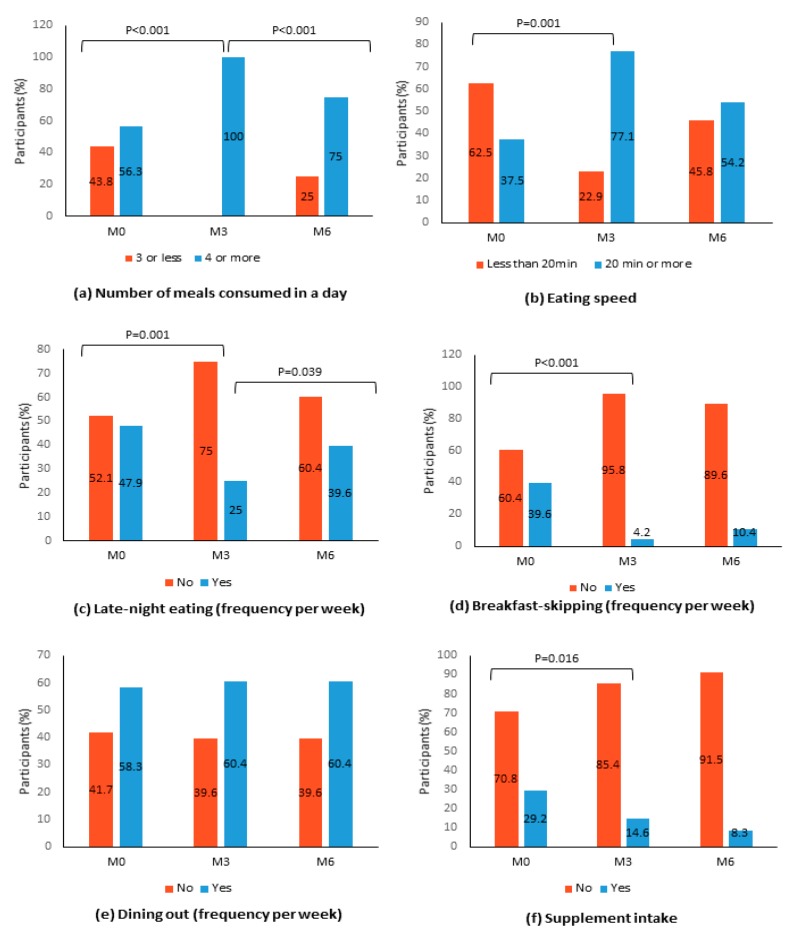
Distribution of study participants according to dietary behaviors at baseline (M0), 3-months post-intervention (M3), and 6-months post-intervention (M6) (N = 48). Note: The McNemar test was performed between baseline (M0) and post-intervention (M3) and between post-intervention (M3) and post-follow-up (M6). Only statistically significant *p* values (*p* < 0.05) are shown.

Changes in the distribution of the peers according to other dietary behaviors were also observed (Figure 2). PERSUADE encouraged participants to consume smaller but more frequent meals to contain hunger. Although the post-intervention finding found all participants adhered to that suggestion (56.3% vs. 100%, *p* < 0.001), the effect was not sustained over the follow-up period. Overall, more peers took longer than 20 minutes to eat their main meals at post-intervention compared to baseline (77.1% vs. 37.5%, *p* = 0.001), though a decline was observed at post-follow-up. Proportions of late-night dining (after 10 pm) and dining out also showed decreases. Although it was initially difficult to discourage late-night food consumption among our participants (52.1% vs. 75%, *p* = 0.001), the proportion of late-night eaters reduced at post follow-up (75% vs. 60.4%, *p* = 0.039). While no statistically significant changes can be observed in dining out behavior, the proportion of participants who skipped breakfast decreased at post-intervention (4.2% vs. 39.6%, *p* < 0.001). As PERSUADE encouraged consumption of real foods compared to supplements, the proportion of peers consuming supplements also reduced from 29.2% to 4.2% at post-intervention (*p* < 0.001). 

### 3.2. Lifestyle Behaviors

**Figure 3:**
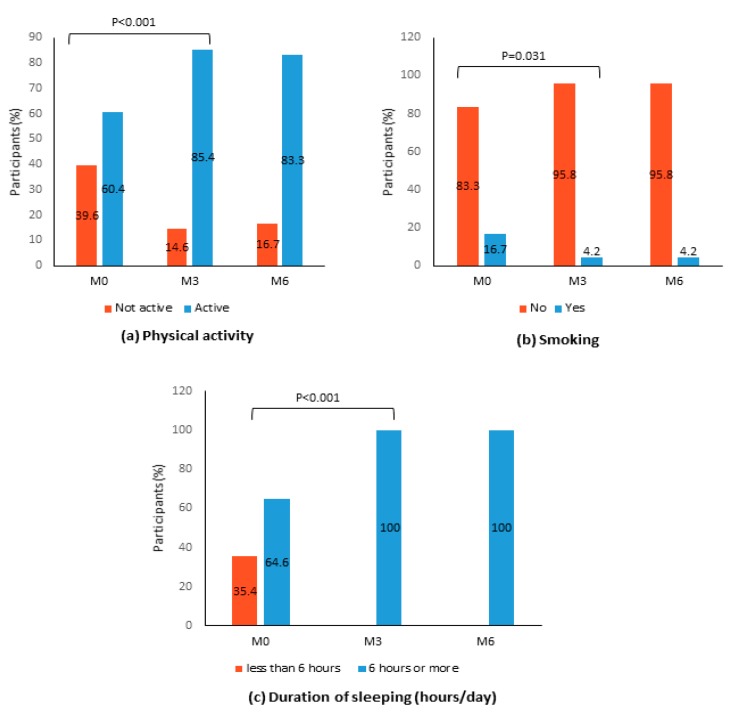
Distribution of study participants according to (**a**) physical activity, (b) smoking and (**c**) sleeping pattern at baseline (M0), 3-months post-intervention (M3), and 6-months post-intervention (M6) (N = 48). Note: The McNemar test was performed between baseline (M0) and post-intervention (M3) and between post-intervention (M3) and post-follow-up (M6). Only statistically significant *p* values (*p* < 0.05) are shown.

A significant increase in the number of physically active peers was reported after 3 months of intervention as compared to baseline (85.4% vs. 60.4%, *p* < 0.001) (Figure 3). Interestingly, we found the proportion of smokers to decline from 16.7% to 4.2% at post-intervention (*p* = 0.031) and remained low at post-follow-up. In addition to this, all participants reportedly had at least 6 hours of sleep per day at post-intervention (*p* < 0.001) and over the follow-up period. 

### 3.3. Anthropometry and Metabolic Parameters

Overall, there were significant changes found in all anthropometry and metabolic parameters, except DBP and BF (Table 3). Pairwise comparisons showed significant differences in SBP, FBG, BMI, and TG between baseline and post-intervention, but not between post-intervention and post-follow-up. A decrease in WC at post-intervention (d = −0.71%, *p* < 0.001) suggested abdominal obesity to be one of the most challenging parameters to be intervened. It was also disappointing to note an increase in WC at post follow-up (d = +0.24, *p* = 0.018). A significant increase observed in HDL at the end of intervention (d = +25.89%, *p* = 0.001) was not retained over the follow-up (d = −19.86%, *p* < 0.001). 

## 4. Discussion

There is growing evidence supporting the adoption of peer support frameworks for the management of chronic non-communicable diseases such as hypertension and T2DM [22,23,24]. The effect of peer support-based lifestyle interventions can be extended to MetS as the clustering of metabolic risk factors predisposes patients to similar metabolic complications in later years. Peer support established a supportive social construct that aims for collective changes to practice a better lifestyle [25]. The effectiveness of peer support is reflected well in the long-term lifestyle changes following continuous social support received by peers [26]. PERSUADE was designed using a systematic approach, taking into consideration the potential for long-term continuous peer support.

PERSUADE resulted in an overall decrease in the fat intake but increased consumption of carbohydrates and protein. The inverse relationship in consumption suggested that participants reduced their intake of fat and re-compensated it with the intake of carbohydrates and protein. However, the overall macronutrient intake was reduced, as reflected by the reduction in total energy intake. Similarly, the PREVENT-DM trial reported significant improvement in dietary habits in a peer-led lifestyle behavior intervention when calorie counting was taught to the peers [27]. PERSUADE demonstrated success in improving dietary behaviors such as late-night eating, slower eating speed, and skipping breakfast. This is an important finding for our peer support program as individual interventions have shown difficulties in achieving notable changes in these aspects and often reported to be successful when social support is included as one of the key intervention features [28]

Although significant changes were observed among critical anthropometric and metabolic parameters, the changes were small in magnitude. Larger magnitudes of change in body weight, for example, were seen in a 24-week peer-led intervention [29], which advises us that there is a need for a longer intervention period in the future. Similarly, PERSUADE had a significant effect on SBP but not DBP, a finding that is consistent with the results from prior studies of peer-led groups where longer periods of intervention may be required to observe changes in DBP [30,31]. 

### 4.1. Lessons Learned from PERSUADE

The success of peer support appears to be due in part to the non-hierarchical, reciprocal relationship that is created through the sharing of similar life experiences [32]. Despite the benefits for patients with chronic diseases from peer support programs in improving psychosocial outcomes for patients [33], the study outlined a few key lessons on what can be improved for a peer support program for MetS.

Peer support provides adults with MetS with better social support. These adults felt more empowered to make collective changes in behavior, which ultimately leads to everyone in peer groups achieving target goals in health and fitness [21]. This is enhanced by a better understanding of MetS acquired by all peers in the early part of PERSUADE. This finding is similar to a previous study reporting that participants’ individual knowledge and attitudes toward MetS risks will significantly improve peer group changes in terms of dietary habits and physical activity [24].

The intervention period of PERSUADE lasted 12 weeks, and we allocated another 12 weeks for follow-up. Feasibility trials with similar duration, small sample size, and study design have been reported in MetS and other conditions [34,35,36]. Greer and Hill [34], for instance, utilized a pre-post study design over a period of 10 weeks to assess healthy lifestyle changes in the individual with MetS (*n* = 22) after interactive group sessions. The study did not find significant statistical changes in weight or BMI but found a slight reduction in WC at week 10.

Consistent and continuous support of peers is essential to ensure significant changes in lifestyle behaviors within a shorter period of intervention (less than 12 weeks) [37]. PERSUADE encouraged the peers to share experiences with one another and learn more about the barriers faced by peers to practice healthy eating and regular physical activity during the 12-weeks intervention period. As a result, the peers learned food portioning and the exact duration of time that can be allocated for their own meal preparations according to their daily activities. Peers were also more engaged in weekly exercise sessions and became proactive enough to initiate group exercise sessions with their peer leaders.

### 4.2. Strengths and Limitations

We expected an increase in the glycemic load as there has been an increase in an overall carbohydrate intake. We hypothesized that the decrease in fat intake was compensated with an increase in carbohydrates and protein. This paradox has been documented in previous literature [38] and happens as the individuals try to compensate for the lower energy intake from reduced fat consumption. Higher intake of carbohydrates is also regarded to be more satiating [39]. In addition, the total sugar intake saw a reduction at post-intervention but increased again at follow-up. The lesson learned here is that a greater emphasis must be given to complex carbohydrate consumption as wholegrain consumption is not the norm in Malaysia. Future studies should also focus on strategies to increase low energy-dense but satiating food options for participants as they are encouraged to lower their fat consumption. 

As this study was focused on the development and test of the feasibility of a peer-led program, two key features lacking in PERSUADE were a control arm and cost-effectiveness analysis. Future studies should include a comparative arm using a randomized-controlled design with a measurement on cost-effectiveness analyses to provide a more in-depth evaluation of a peer support program such as PERSUADE. Since the cost of managing chronic diseases is continuously increasing, it is relevant to determine the cost-effectiveness of the peer support framework as compared to the current standard treatment.

Peer-led interventions in the future should also incorporate local community stakeholders to increase the participation rate and crucial continuous support such as regular health check programs, regular exercise events, and events with community healthcare clinics. Peer support approaches such as face-to-face, telephone contact, or web-based/email can also be incorporated into the intervention model to improve the outcomes.

## 5. Conclusions

PERSUADE is a peer-led intervention program for individuals with a risk of MetS that shows a positive result in its pre-post feasibility trial. In PERSUADE pre-post trial, 12 weeks of intervention resulted in significant improvements in vital nutrition and lifestyle behaviors, as well as small yet promising improvements in anthropometric and metabolic parameters. 

Future work entails implementation in a larger sample and a control group, with low energy-dense, satiating foods or wholegrain alternatives, the involvement of other stakeholders in program development and delivery, as well as the use of various intervention modalities to improve the nutritional and metabolic outcomes. 

## Figures and Tables

**Table 1:** Characteristics of study participants (N = 48).

Characteristics		All (N = 48)	PG1 (*n* = 14)	PG2 (*n* = 10)	PG3 (*n* = 15)	PG4 (*n* = 9)	*p*
Age (Years)	Median (IQR)	46 (11)	43.5 (11)	47 (6)	44 (17)	46 (7)	0.563
Gender	Female	25 (52.1)	6 (42.9)	6 (60.0)	6 (40.0)	7 (77.8)	0.292
	Male	23 (47.9)	8 (57.1)	4 (40.0)	9 (60.0)	2 (22.2)	
Ethnicity	Malay	41 (85.4)	12 (85.7)	9 (90.0)	13 (86.7)	7 (77.8)	0.515
	Chinese	3 (6.3)	1 (7.1)	0 (0.0)	2 (13.3)	0 (0.0)	
	Indian	4 (8.3)	1 (7.1)	1 (10.0)	0 (0.0)	2 (22.2)	
Marital status	Single	2 (4.2)	1 (7.1)	0 (0.0)	1 (6.7)	0 (0.0)	0.967
	Married	44 (91.7)	13 (92.9)	9 (90.0)	13 (86.7)	9 (100.0)	
	Widowed	2 (4.2)	0 (0.0)	1 (10.0)	1 (6.7)	0 (0.0)	
Education	Primary	5 (10.4)	1 (7.1)	1 (10.0)	1 (6.7)	2 (22.2)	0.658
	Lower secondary	13 (27.1)	2 (14.3)	4 (40.0)	3 (20.0)	4 (44.4)	
	Upper secondary	16 (33.3)	6 (42.9)	3 (30.0)	5 (33.3)	2 (22.2)	
	Tertiary	14 (29.2)	5 (35.7)	2 (20.0)	6 (40.0)	1 (11.1)	
Occupation	Working	47 (97.9)	13 (92.9)	10 (100.0)	15 (100.0)	9 (100.0)	0.687
	Not working	1 (2.1)	1 (7.1)	0 (0.0)	0 (0.0)	0 (0.0)	

PG = peer group. Age is presented as median (IQR) and analyzed with the Kruskal Wallis test. Categorical variables are presented as *n* (%) and were analyzed with Fisher’s Exact test.

**Table 2:** Changes in nutrient intake of study participants (N = 48).

	Baseline (M0)	Post-Intervention (M3)	Post-Follow-Up (M6)	*p* ^a^	Pairwise Comparison	Change (%)	*p* ^b^
	Mean (SD)	Mean (SD)	Mean (SD)				
Energy (kcal)	1685.42 (421.04)	1613.82 (331.50)	1453.24 (296.61)	0.001	M0 vs. M3	−4.25	0.045
					M3 vs. M6	−9.90	0.015
Carbohydrate (g/1000 kcal)	139.71 (32.35)	149.04 (42.91)	165.19 (33.21)	0.001	M0 vs. M3	−6.68	0.483
					M3 vs. M6	10.84	0.049
Protein (g/1000 kcal)	32.91 (9.53)	33.33 (14.54)	39.93 (13.71)	0.004	M0 vs. M3	−1.19	1.000
					M3 vs. M6	+19.80	0.041
Fat (g/1000 kcal)	29.03 (11.89)	21.89 (12.44)	22.96 (9.81)	0.001	M0 vs. M3	−24.60	<0.001
					M3 vs. M6	+4.89	1.000
Total fiber ^c^ (g/1000 kcal)	6.71 (2.05)	6.95 (6.60)	9.06 (3.41)	<0.001	M0 vs. M3	+3.58	0.508
					M3 vs. M6	+30.36	<0.001
Total sugar (g/1000 kcal)	40.02 (18.64)	32.05 (17.00)	44.57 (20.42)	0.001	M0 vs. M3	−24.87	0.056
					M3 vs. M6	+39.06	0.002
Glycemic load (g/%)	135.34 (30.05)	157.54 (47.22)	156.43 (44.16)	<0.001	M0 vs. M3	+14.93	<0.001
					M3 vs. M6	−0.70	1.000

^a^ Repeated measures; ^b^ Bonferroni pairwise post hoc; ^c^ Natural log transformation was performed.

**Table 3:** Changes in anthropometry and metabolic parameters of study participants (N = 48).

	Baseline (M0)	Post-Intervention (M3)	Post Follow-Up (M6)	*p* ^a^	Pairwise Comparison	Change (%)	*p* ^b^
	Mean (SD)	Mean (SD)	Mean (SD)				
SBP (mmHg)	135.29 (19.65)	130.50 (17.36)	130.42 (18.36)	0.001	M0 vs. M3	−3.54	0.001
					M3 vs. M6	−0.06	1.000
DBP (mmHg)	82.58 (11.67)	81.00 (9.28)	81.75 (9.67)	0.566			
		
FBG (mmol/L)	8.60 (3.48)	7.57 (1.98)	7.57 (2.16)	<0.001	M0 vs. M3	−11.98	<0.001
					M3 vs. M6	0	1.000
BMI (kg/m^2^)	25.84 (3.91)	25.42 (3.93)	25.50 (4.07)	0.001	M0 vs. M3	−1.63	<0.001
					M3 vs. M6	+0.31	1.000
WC (cm)	91.72 (11.53)	91.07 (11.36)	91.29 (11.43)	<0.001	M0 vs. M3	−0.71	<0.001
					M3 vs. M6	+0.24	0.018
BF (%)	29.88 (6.57)	29.42 (6.43)	29.37 (6.36)	0.060			
		
TG (mmol/L)	2.89 (1.73)	2.19 (2.06)	2.19 (2.09)	<0.001	M0 vs. M3	−24.22	<0.001
					M3 vs. M6	0	1.000
HDL (mmol/L)	1.12 (0.35)	1.41 (0.32)	1.13 (0.33)	<0.001	M0 vs. M3	25.89	0.001
					M3 vs. M6	−19.86	<0.001

SBP = systolic blood pressure; DBP = diastolic blood pressure; FBG = fasting blood glucose; BMI= body mass index; WC = waist circumference; BF = body fat; TG = triglyceride; HDL= high-density lipoprotein cholesterol. ^a^ Repeated measures; ^b^ Bonferroni pairwise post hoc.
